# Opioid-free versus opioid-based anesthesia in laparoscopic bariatric surgery: an exploratory randomized pilot trial

**DOI:** 10.1186/s12871-026-03838-4

**Published:** 2026-05-25

**Authors:** Emanuel Almeida, Fábio Almeida, Carina Rossoni, Rui Ribeiro

**Affiliations:** 1 Multidisciplinary Center for the Treatment of Obesity and Diabetes at Lusíadas Hospital Amadora, Amadora, Portugal; 2 Faculty of Medicine, Institute of Environmental Health, University of Lisbon, Lisbon, Portugal; 3 Multidisciplinary Center for the Treatment of Obesity and Diabetes at Lusíadas Hospital Lisbon, Lisbon, Portugal

**Keywords:** Severe obesity, Anesthesia, Opioids, Bariatric surgery, Metabolic surgery, Postoperative pain, PONV

## Abstract

**Background:**

Bariatric surgery is the most effective treatment for severe obesity, but perioperative pain control and prevention of postoperative nausea and vomiting (PONV) remain important clinical challenges. Opioid-free anesthesia (OFA) has been proposed as a strategy to reduce opioid-related adverse effects within multimodal perioperative care; however, evidence regarding its clinical benefits in bariatric surgery remains limited and heterogeneous.

**Methods:**

In this prospective exploratory randomized pilot trial, 60 patients undergoing laparoscopic bariatric surgery were allocated in a 1:1 ratio to receive opioid-free anesthesia (OFA) or opioid-based anesthesia (OBA). The primary endpoint was postoperative pain intensity at post-anesthesia care unit (PACU) discharge. Secondary outcomes included pain at additional time points, need for rescue analgesia, PONV, intraoperative nociception assessed using the nociception level (NOL) index, and patient satisfaction. Adjusted analyses were performed using generalized linear and logistic regression models, accounting for clinically relevant baseline imbalances.

**Results:**

Sixty patients were randomized (OFA *n* = 30; OBA *n* = 30), with no losses to follow-up. OFA was associated with lower postoperative pain scores at PACU discharge and at 24 h, but not at hospital discharge. Although absolute differences in pain intensity were modest, the need for rescue analgesia was lower in the OFA group (30.0% vs. 73.3%), corresponding to an absolute risk reduction of 43.3% and a number needed to treat of approximately 3. Adjusted analyses were consistent with these findings, showing higher odds of requiring rescue analgesia in the OBA group (adjusted OR 6.5, 95% CI 1.8–23.2), although the wide confidence interval reflects considerable imprecision. The incidence of PONV was similar between groups, although interpretation is limited by unequal antiemetic prophylaxis. Intraoperative nociception, assessed using the NOL index in a subset of patients, showed lower values in the OFA group, although these findings are exploratory.

**Conclusions:**

OFA was associated with modest reductions in postoperative pain and a clinically meaningful decrease in rescue analgesia requirements. These findings suggest that OFA may represent a promising opioid-sparing strategy but should be considered hypothesis-generating and require confirmation in larger, adequately powered multicentre randomized trials.

**Trial registration:**

NCT07337135. Registered on 13th January 2026. Retrospectively registered.

## Introduction

Obesity is a chronic disease affecting approximately 890 million people worldwide [1] and is associated with a range of serious comorbidities, including type 2 diabetes mellitus (T2DM), arterial hypertension (HTN), obstructive sleep apnea syndrome (OSAS) and obesity hypoventilation syndrome (OHS) and metabolic syndrome. The latter is defined by the presence of three of the following five criteria: waist circumference > 102 cm (men) or > 88 cm (women); triglycerides > 150 mg/dL; HDL cholesterol < 40 mg/dL (men) or < 50 mg/dL (women); blood pressure ≥ 130/85 mmHg; fasting glucose > 100 mg/dL [1].

Bariatric surgery is widely recognized as the most effective intervention for sustained weight loss, control of obesity-related comorbidities, and improvement in life expectancy in severe cases [2–5]. Approximately 600,000 patients undergo bariatric surgery annually, with an even larger population meeting criteria for the procedure [2].

The most commonly reported perioperative complications include deep vein thrombosis (DVT) / pulmonary embolism (PE), anastomotic leak, respiratory failure, hemorrhage, and surgical site infection [3].

In high-volume reference centers, bariatric surgery carries a 30-day mortality rate of 0.5% and a morbidity rate of 5% [3].

Over recent years, the Enhanced Recovery After Surgery (ERAS) program has gained worldwide adoption across multiple surgical specialties. This program — consisting of a series of evidence-based, multidisciplinary perioperative interventions primarily aimed at improving postoperative recovery — has been shown to reduce morbidity, hospital length of stay, and associated costs, while increasing patient satisfaction [5]. The first ERAS guidelines for perioperative management in bariatric surgery were published in 2016 and later updated in 2021 to reflect evolving evidence [5].

Despite these updates, current scientific evidence still does not allow the recommendation of a specific anesthetic technique or specific anesthetic agents. However, there is consensus that the use of rapidly reversible, fast-acting drugs contributes to improved postoperative recovery [5]. Within ERAS pathways, minimizing opioid exposure while maintaining effective analgesia remains a key perioperative objective.

In opioid-based anesthesia (OBA), traditionally used to achieve antinociception, remifentanil is the drug of choice. Nevertheless, rapid tolerance to opioids, along with a well-known profile of postoperative adverse effects (nausea and vomiting, respiratory depression, prolonged sedation, urinary retention, ileus, postoperative hyperalgesia, and chronic pain) [6], may prolong hospitalization or even result in unplanned readmissions.

Recently, there has been a growing shift toward opioid-free anesthesia (OFA), focusing on the use of adjuvant drugs (lidocaine, dexmedetomidine, ketamine, etc.) and regional anesthesia to achieve hypnosis, amnesia, and hemodynamic stability, while avoiding the adverse effects associated with opioids. This approach has demonstrated a more favorable profile, contributing to earlier return of bowel motility, reduced postoperative hyperalgesia, and lower incidence of postoperative nausea and vomiting (PONV) [5].

However, randomized evidence comparing OFA and OBA in bariatric surgery remains limited, particularly regarding clinically relevant postoperative outcomes such as pain, opioid consumption, and PONV.

Given these considerations, the objective of this study was to compare the efficacy of opioid-based anesthesia (OBA) versus opioid-free anesthesia (OFA) in patients undergoing laparoscopic bariatric surgery. We hypothesized that the OFA technique would result in improved pain control, lower incidence of PONV, and greater overall patient satisfaction.

## Methods

This was a prospective, exploratory randomized pilot trial including 60 patients undergoing laparoscopic bariatric surgery at Hospital Lusíadas Amadora, Portugal, a private multidisciplinary hospital with intensive care capability and an established bariatric surgery program. Ethical approval was obtained from the Institutional Ethics Committee on 17th July 2023. Patients were consecutively recruited between 10th October 2023 and 19th April 2024, and all participants provided written informed consent prior to inclusion. Participants were followed from the intraoperative period until hospital discharge, with outcomes assessed intraoperatively, in the PACU, within the first 24 postoperative hours, and at discharge. Inclusion criteria were: age ≥ 18 years; body mass index (BMI) ≥ 35 kg/m² with associated comorbidities, or BMI ≥ 40 kg/m². Exclusion criteria were: pregnancy, history of substance abuse, or severe psychiatric disease.

No formal a priori sample size calculation was performed, as this study was designed as an exploratory randomized pilot trial. The sample size was determined by the number of consecutive eligible patients recruited during the predefined study period, resulting in the inclusion of 60 participants. The primary aim was to assess feasibility and to generate preliminary effect size estimates for postoperative pain outcomes, which may inform the sample size calculation of a future adequately powered randomized trial. Secondary outcomes were exploratory and not powered for definitive comparisons but were included to provide clinically relevant effect estimates for future studies.

**Fig. 1:**
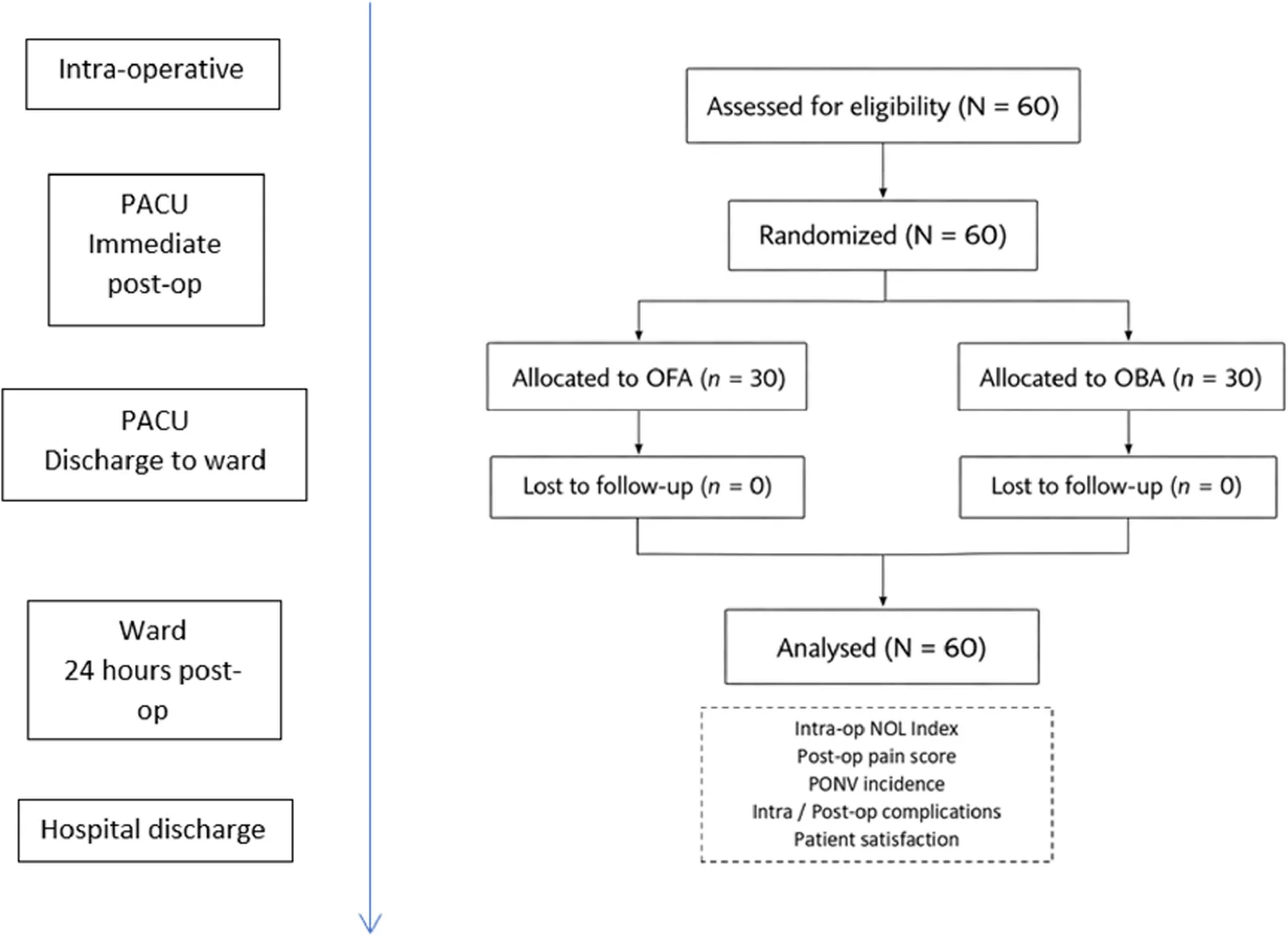
CONSORT flow diagram

Patients were randomized in a 1:1 ratio to receive either opioid-free anesthesia (OFA) or opioid-based anesthesia (OBA) (Fig. 1). The random allocation sequence was generated by an investigator not involved in recruitment or clinical management. Allocation concealment was ensured using sequentially prepared assignments opened only at the time of inclusion.

Participants were blinded to treatment allocation. Blinding was facilitated by the use of general anesthesia with similar perioperative management in both groups, and the anesthetic protocol was not disclosed to patients. Due to the nature of the intervention, blinding of anesthesiologists and PACU staff was not feasible, as the continuation of postoperative infusions in the OFA group could reveal group assignment. Consequently, outcome assessment was not blinded. Statistical analysis was performed by an independent investigator blinded to treatment allocation.

A CONSORT flow diagram describing participant enrolment, allocation, follow-up, and analysis is presented in Fig. 1.

Each participant underwent an anesthetic intervention according to the respective protocol (Table 1):

**Table 1:** Anesthetic protocols

OFA group	OBA group
Standard monitoring + BIS + TOF + NOL Venous catheterization and initiation of goal-directed fluid therapy with balanced crystalloid Bolus of dexmedetomidine 15–20 µg followed by infusion: dexmedetomidine 50 µg + ketamine 50 mg + lidocaine 500 mg in 50 mL saline (rate: 1 mL / 10 kg total body weight) Induction with propofol Neuromuscular blockade with rocuronium Maintenance with desflurane PONV prophylaxis with dexamethasone 4–8 mg after induction + ondansetron 30 min before the end of the surgery Antibiotic prophylaxis with cefazolin 2–4 g Stress-ulcer prophylaxis with esomeprazole 40 mg Postoperative analgesia: paracetamol + ketorolac/parecoxib + metamizole Infusion rate reduced by half 30 min before the end of surgery (continued for 1 h in PACU, until completion or discharge) Reversal of neuromuscular blockade with sugammadex Rescue analgesia in PACU with tramadol 100 mg PONV treatment in PACU with ondansetron 4 mg	Standard monitoring + BIS + TOF + NOL Venous catheterization and initiation of goal-directed fluid therapy with balanced crystalloid Start of remifentanil infusion (2 mg / 40 mL saline) Induction with propofol Neuromuscular blockade with rocuronium Maintenance with desflurane PONV prophylaxis with dexamethasone 4–8 mg after induction + ondansetron 4 mg + droperidol 0,625 mg 30 min before the end of the surgery Antibiotic prophylaxis with cefazolin 2–4 g Stress-ulcer prophylaxis with esomeprazole 40 mg Postoperative analgesia: paracetamol + ketorolac/parecoxib + metamizole Termination of remifentanil infusion at the end of surgery Reversal of neuromuscular blockade with sugammadex Rescue analgesia in PACU with tramadol 100 mg and/or morphine PONV treatment in PACU with ondansetron 4 mg

Both anesthetic protocols were administered by experienced anesthesiologists according to the predefined study protocol, and all patients received the allocated intervention as intended. No relevant deviations from the planned interventions were recorded.

Apart from the anesthetic strategy, perioperative care was standardized across both groups, including monitoring, fluid therapy, antibiotic prophylaxis, and multimodal postoperative analgesia. In the OFA group, intraoperative infusions of dexmedetomidine, lidocaine, and ketamine were continued into the early postoperative period and maintained for approximately 1 h in the PACU as part of the multimodal analgesic strategy. Antiemetic prophylaxis was administered according to routine clinical practice.

Given the anticipated higher risk of PONV associated with opioid use in bariatric patients, a more intensive prophylactic regimen was used in the OBA group. All surgical procedures were performed by the same bariatric surgical team.

The primary outcome was postoperative pain intensity at discharge from the post-anesthesia care unit (PACU), assessed using the Numerical Rating Scale (NRS; 0–10) [7]. Secondary outcomes included postoperative pain at additional predefined time points (within 30 min of PACU admission, at 24 h, and at hospital discharge), the need for rescue analgesia (with or without opioids), the incidence of postoperative nausea and vomiting (PONV) and the need for pharmacological treatment, intraoperative nociception assessed using the Nociception Level Index (NOL; Medasense^®^), and patient satisfaction measured on a 1–10 numerical scale.

The following variables were collected to characterize the study population and clinical course. Demographic variables included age and sex. Anthropometric and clinical variables comprised weight, height, and body mass index (BMI), as well as comorbidities (hypertension, type 2 diabetes mellitus, and obstructive sleep apnea syndrome/obesity hypoventilation syndrome [OSAS/OHS]), regular medication use (including β-blockers, other antihypertensive agents, statins, glucocorticoids, synthetic opioids, antidepressants, and other medications), and alcohol and tobacco consumption.

Surgical variables included the type of procedure performed, surgical duration, intraoperative and postoperative complications, and length of hospital stay. Anesthetic variables comprised intraoperative nociception assessed using the NOL index, postoperative pain intensity measured using the NRS at predefined time points (within 30 min of PACU admission, at PACU discharge [up to 2 h after admission], within the first 24 postoperative hours, and at hospital discharge), the need for rescue analgesia, and PONV-related outcomes, including incidence, need for treatment, and severity assessed using the PONV Impact Scale at 6 and 24 h postoperatively [8].

Biochemical variables included hemoglobin, leukocyte count, C-reactive protein (CRP), procalcitonin, and erythrocyte sedimentation rate (ESR).

Adverse events were recorded if they occurred during the intraoperative period or PACU stay, based on routine clinical documentation in the anesthetic and recovery records. No predefined safety endpoints or systematic screening protocol were implemented.

Statistical analysis was performed using IBM SPSS Statistics (version 28.0; IBM Corp., Armonk, NY, USA).

Continuous variables were assessed for normality using the Shapiro–Wilk test and are presented as mean ± standard deviation (SD) or median and interquartile range (IQR), as appropriate. Categorical variables are presented as counts and percentages. Baseline characteristics were described without formal statistical comparisons, in accordance with CONSORT recommendations. Absolute standardized differences (ASD) were calculated to assess baseline balance between groups. An ASD threshold of > 0.20 was used to identify potentially important imbalance. To preserve model stability, variables with an absolute frequency of fewer than 5 observations in either group were not included in adjusted models regardless of ASD value.

The primary endpoint, postoperative pain intensity at PACU discharge, and other continuous outcomes were analyzed using generalized linear models (GLMs), with model specification based on outcome distribution (identity link for approximately normally distributed outcomes; Tweedie distribution with log link for skewed outcomes). Dichotomous outcomes, including the need for rescue analgesia, postoperative nausea and vomiting (PONV), and requirement for antiemetic treatment, were analyzed using logistic regression models. To account for baseline imbalances while preserving degrees of freedom in this exploratory pilot sample, a propensity score was estimated from covariates showing important imbalance (ASD > 0.20 and adequate frequency) and included as an adjustment covariate in multivariable models. Intraoperative nociception (NOL index) was analyzed descriptively. Exploratory analyses of categorized NOL values at predefined intraoperative time points were performed using logistic regression in patients with available data. Effect sizes are reported as regression coefficients (b) for continuous outcomes and odds ratios (OR) or risk ratios (RR), as appropriate, with 95% confidence intervals (CI).

Given the exploratory pilot design, analyses focused on effect size estimation. Secondary outcomes were considered exploratory. All randomized participants were analyzed according to the intention-to-treat principle. There were no missing data; all analyses were performed on complete cases.

A two-sided p-value < 0.05 was considered statistically significant. Bonferroni correction was applied for multiple comparisons where appropriate. No interim analyses were performed.

## Results

A total of 60 patients were randomized in a 1:1 ratio to receive OFA or OBA. No participants were lost to follow-up, and no exclusions occurred after randomization. All randomized patients completed the study and were included in the final analysis.

The study population (*n* = 60) was predominantly female (76.7%), with a mean age of 44.9 ± 10.6 years and a mean BMI of 39.1 ± 5.0 kg/m². Hypertension was the most frequent comorbidity (36.7%), followed by dyslipidemia and depressive syndrome. Baseline demographic and clinical characteristics are presented descriptively by group in Table 2, in accordance with CONSORT recommendations. No formal statistical comparisons between groups were performed. Absolute standardized differences (ASD) were calculated to assess baseline balance. Several variables showed moderate imbalance (ASD > 0.20), which is plausible given the small sample size of this exploratory randomized pilot trial. To reduce potential confounding arising from these residual imbalances, adjusted analyses were performed using a propensity score derived from clinically relevant baseline covariates meeting the prespecified imbalance criteria.

**Table 2:** Baseline characteristics of patients in the OFA and OBA groups (*n* = 60)

Characteristics*	OFA (*n* = 30)	OBA (*n* = 30)	ASD**
Age (years)	47.2 ± 10.3	42.7 ± 10.5	0.43
Sex			
Male, n (%)	8 (26.7)	6 (20.0)	0.16
Female, n (%)	22 (73.3)	24 (80.0)	
BMI (kg/m^2^)	39.3 ± 5.0	39.0 ± 5.0	0.08
Comorbidities			
Hypertension, n (%)	13 (43.3)	9 (30.0)	0.28
Dyslipidemia, n (%)	12 (40.0)	8 (26.7)	0.28
Type 2 Diabetes, n (%)	4 (13.3)	3 (10.0)	0.10
OSAS/OHS, n (%)	7 (23.3)	6 (20.0)	0.08
Depression / antidepressant use, n (%)	13 (43.3)	6 (20.0)	0.52
Smoking, n (%)	5 (16.7)	7 (23.3)	0.17
Alcohol use, n (%)	13 (43.3)	18 (60.0)	0.34
Type of surgery, n (%)			
Sleeve gastrectomy	1 (3.3)	1 (3.3)	0.00
Gastric bypass	27 (90)	26 (86.7)	0.1
Revisional bypass	2 (6.7)	3 (10)	0.13

* Data are presented as mean ± standard deviation or n (%). ** Absolute standardized differences (ASD) were calculated to assess baseline balance, with values > 0.20 considered indicative of potentially important imbalance

The distribution of surgical procedures was similar between groups. Sleeve gastrectomy was performed in two patients (one per group), whereas the remaining procedures were gastric bypass. Among bypass procedures, five were revisional surgeries, with a comparable distribution between groups (OBA *n* = 3; OFA *n* = 2).

Intraoperative nociception monitoring using the NOL index was available in 25 of 60 patients (41.7%). At 15 min, higher odds of elevated NOL values were observed in the OBA group (OR 7.7, 95% CI 1.16–51), with a similar direction after adjustment (adjusted OR 21.9, 95% CI 1.4–330), although the wide confidence intervals indicate substantial imprecision. At subsequent time points, lower NOL values were generally observed in the OFA group; however, these findings should be considered exploratory, given the limited sample size and wide confidence intervals, as well as the absence of NOL-guided intraoperative management (Table 3).

**Table 3:** Intraoperative nociception (NOL Index) in the OFA and OBA groups (*n* = 60)

Variables*	OFA (*n* = 30)	OBA (*n* = 30)
NOL Measurement Availability		
Measured	13 (43.3)	12 (40.0)
Not Measured	17 (56.7)	18 (60.0)
NOL Index		
15 min	13 (100)	12 (100)
30 min	13 (100)	12 (100)
45 min	13 (100)	12 (100)
60 min	13 (100)	11 (91.7)
75 min	13 (100)	10 (83.3)
90 min	11 (84.6)	9 (75.0)
105 min	8 (61.5)	7 (58.3)
120 min	8 (61.5)	6 (50.0)
135 min	7 (53.8)	3 (27.3)
150 min	1 (7.7)	2 (16.7)
165 min	1 (7.7)	1 (8.3)
180 min	1 (7.7)	1 (8.3)
NOL Index 15 min		
< 10	11 (84.6)	5 (41.7)
10–25	2 (15.4)	7 (58.3)
NOL Index 30 min		
< 10	12 (92.3)	6 (50.0)
10–25	1 (7.7)	4 (33.3)
26–45	0 (0.0)	2 (16.7)
NOL Index 45 min		
< 10	11 (84.6)	10 (83.3)
10–25	2 (15.4)	1 (8.3)
26–45	0 (0.0)	1 (8.3)
NOL Index 60 min		
< 10	13 (100)	8 (72.7)
10–25	0 (0.0)	2 (18.2)
26–45	0 (0.0)	1 (9.1)
NOL Index 75 min		
< 10	12 (92.3)	8 (80.0)
10–25	1 (7.7)	1 (10.0)
26–45	0 (0.0)	1 (10.0)
NOL Index 90 min		
< 10	10 (90.9)	5 (55.6)
10–25	1 (9.1)	3 (33.3)
26–45	0 (0.0)	1 (11.1)
NOL Index 105 min		
< 10	1 (100)	0 (0.0)
10–25	0 (0.0)	1 (100)
26–45	0 (0.0)	1 (14.3)
NOL Index 120 min		
< 10	7 (87.5)	3 (50.0)
10–25	1 (12.5)	3 (50.0)
NOL Index 135 min		
< 10	6 (85.7)	2 (66.7)
10–25	1 (14.3)	1 (33.3)
NOL Index 150 min		
< 10	1 (100)	1 (50.0)
10–25	0 (0.0)	1 (50.0)
NOL Index 165 min		
< 10	1 (100)	1 (100)
NOL Index 180 min		
< 10	1 (100)	0 (0.0)
10–25	0 (0.0)	1 (100)

* Data are presented descriptively; no formal statistical comparisons are reported

Postoperative outcomes are presented descriptively in Table 4. Postoperative pain scores were lower in the OFA group across recovery. Rescue analgesia was required in 30.0% (9/30) of patients in the OFA group compared with 73.3% (22/30) in the OBA group, corresponding to an absolute risk reduction of 43.3% and a number needed to treat of approximately 3.

**Table 4:** Postoperative pain during hospitalization in OFA and OBA groups (*n* = 60)

Characteristics	OFA (*n* = 30)	OBA (*n* = 30)
Postoperative Pain (NRS)		
PACU Admission	0 (0–2)	2.5 (0–5.5)
PACU Discharge	1 (0–3)	3 (1–5)
First 24 h	1 (0–3)	3 (2–4)
Hospital Discharge	0 (0–0)	0 (0–2)
Need for rescue analgesia		
Yes	9 (30.0)	22 (73.3)
No	21 (70.0)	8 (26.7)
Use of opioids		
Yes	8 (88.9)	21 (95.5)
No	1 (11.1)	1 (4.5)

* Data are presented as median (interquartile range). Descriptive comparisons are provided; formal statistical inference is reported in adjusted analyses (Table 5)

Adjusted analyses using generalized linear models (Table 5) showed reductions in postoperative pain at PACU discharge (adjusted b − 1.1, 95% CI − 2.1 to − 0.1) and at 24 h (adjusted b − 1.0, 95% CI − 2.0 to − 0.03), but not at hospital discharge (adjusted b − 0.4, 95% CI − 1.2 to 0.3; *p* = 0.263). Adjusted logistic regression analysis incorporating the propensity score showed higher odds of requiring rescue analgesia in the OBA group (OR 6.5, 95% CI 1.8–23.2).

**Table 5:** Adjusted associations between anesthetic technique and postoperative outcomes

Variables*	OFA (*n* = 30)	OBA (*n* = 30)	Difference (CI 95%)	OR (CI 95%)	Adjusted OR/b** (CI 95%)	*p*
NOL Index 15 min						
• 10–25	2 (15.4)	7 (58.3)	-42.9 (-77.0 to -8.8)	7.7 (1.16 to 51)	21.9 (1.4 to 330)	0.026
Postoperative Pain						
• PACU Discharge	1 (0–3)	3 (1–5)	-0.8 (-1.2 to -0.3)	-	-1.1 (-2.1 to -0.1)	0.039
• First 24 h	1 (0–3)	3 (2–4)	-0.4 (-0.8 to -0.1)	-	-1.0 (-2.0 to -0.03)	0.044
• Hospital Discharge	0 (0–0)	0 (0–2)	-1.0 (-1.9 to 0.0)	-	-0.4 (-1.2 to 0.3)	0.263
Need for rescue analgesia	9 (30.0)	22 (73.3)	-43.3 (-66.1 to -20.5)	6.4 (2.1 to 19.8)	6.5 (1.8 to 23.2)	0.004
PONV Impact Scale Score						
• 6 h postop: ≥ 1	8 (26.7)	8 (26.7)	0.0 (-22.4 to 22.4)	1.0 (0.3 to 3.1)	0.6 (0.2 to 2.0)	0.375
• 24 h postop: ≥ 1	3 (10.0)	4 (13.3)	-3.3 (-19.6 to 12.9)	1.4 (0.3 to 6.8)	0.6 (0.1 to 4.0)	0.634
Patient satisfaction	9.3 ± 1.2	9.0 ± 1.2	0.3 (-0.3 to 0.9)	-	0.3 (-0.3 to 0.8)	0.351

* Data are presented as median (25th − 75th percentiles), n (%), or mean ± standard deviation. ** Estimates are adjusted for the propensity score. For categorical outcomes, odds ratios (ORs) with 95% confidence intervals (CIs) are reported; for continuous outcomes, adjusted regression coefficients (b) with 95% CIs are reported to estimate the magnitude of the effect of anesthetic technique

No meaningful differences were observed in adjusted analyses for PONV outcomes. PONV incidence and severity were similar between groups at 6 and 24 h postoperatively (Table 6), and the need for pharmacological treatment was comparable (5/30 [16.7%] in OFA vs. 4/30 [13.3%] in OBA; OR 1.30, 95% CI 0.32–5.27).

**Table 6:** PONV occurrence in OFA vs. OBA Groups (*n* = 60)

Characteristics	OFA (*n* = 30)	OBA (*n* = 30)
	*n* (%)	*n* (%)
PONV Impact Scale Score 6 h PO		
0	22 (73.3)	22 (73.3)
1	4 (13.3)	3 (10.0)
2	3 (10.0)	4 (13.3)
≥ 3	1 (3.3)	1 (3.3)
PONV Impact Scale Score 24 h PO		
0	27 (90.0)	26 (86.7)
1	1 (3.3)	1 (3.3)
2	2 (6.7)	1 (3.3)
≥ 3	0 (0.0)	2 (6.7)
Need for PONV treatment		
Yes	5 (16.7)	4 (13.3)
No	25 (83.3)	26 (86.7)

*Data are presented descriptively; no formal statistical comparisons are reported

Patient satisfaction was high in both groups, with no statistically significant differences observed between groups in adjusted analyses.

Safety outcomes were not systematically collected; therefore, no conclusions can be drawn regarding the safety profile of the interventions.

## Discussion

In this exploratory randomized pilot trial, opioid-free anesthesia was associated with consistently lower postoperative pain scores and a reduced need for rescue analgesia compared with opioid-based anesthesia. Although absolute differences in pain intensity were modest (approximately 1–2 NRS points), they were accompanied by a clinically meaningful reduction in additional analgesic requirements. Rescue analgesia was required in 30.0% of patients in the OFA group compared with 73.3% in the OBA group (risk ratio (RR) 0.41, 95% CI 0.23–0.74; absolute risk reduction 43.3%; corresponding to a number needed to treat of approximately 3).

The reduction in the need for rescue analgesia represents the most clinically relevant finding of this study. Patients in the OFA group were substantially less likely to require additional postoperative analgesia, with adjusted analyses suggesting higher odds of rescue analgesia in the OBA group (OR 6.5, 95% CI 1.8–23.2). Although the confidence interval reflects considerable imprecision due to the limited sample size, the consistency between adjusted and unadjusted analyses is aligned with this finding. These results suggest that the principal clinical benefit of OFA may relate more to reduced postoperative analgesic requirements than to large differences in reported pain intensity.

Although reductions in pain scores were modest (approximately 1–2 NRS points), they were consistent across early postoperative time points and accompanied by a clinically meaningful reduction in rescue analgesic requirements. Notably, differences were no longer apparent at hospital discharge, suggesting that the benefit of OFA may be more pronounced in the early postoperative period. This pattern may reflect the routine use of multimodal non-opioid analgesia in both groups, which likely reduced overall pain levels and limited the magnitude of detectable between-group differences. Additionally, the higher use of rescue analgesia in the OBA group may have attenuated pain score differences over time, as pain assessments were performed after rescue medication when required.

Because several baseline variables showed residual imbalance despite randomization, a propensity score–based adjustment strategy was used to account for these differences while preserving degrees of freedom in this small exploratory sample. Adjusted analyses showed effect estimates in the same direction as unadjusted analyses, supporting the overall consistency of the observed associations. Overall, these findings provide clinically interpretable effect size estimates that may inform the design of future adequately powered randomized trials.

In our cohort, the reduction in analgesic requirements extended beyond the immediate postoperative period. One possible explanation is the continuation of co-analgesic infusions into the early recovery phase. In many previous studies, OFA protocols are discontinued at the end of surgery, potentially limiting their postoperative impact [9, 10] . In contrast, in the present study, the OFA regimen was maintained into the early postoperative period, which may have contributed to sustained modulation of nociceptive processing.

Similar patterns have been described in the literature, where small but consistent reductions in pain scores did not always translate into large clinical effects. Meta-analytic data in bariatric surgery have shown modest reductions in pain intensity with OFA, often without substantial differences in opioid consumption beyond the immediate postoperative period [11–13] . In contrast, in our study, relatively small reductions in pain intensity were accompanied by a lower need for rescue analgesia, suggesting a clinically relevant analgesic-sparing effect associated with the OFA protocol. Taken together, these findings support the interpretation that OFA may primarily reduce postoperative analgesic requirements, with more limited effects on perceived pain intensity.

With regard to intraoperative nociception, NOL monitoring was available in a subset of patients and was not used to guide analgesic management. Although lower values were generally observed in the OFA group, these findings should be interpreted with caution given the limited sample size and incomplete data availability. An association at the 15-minute time point was observed; however, effect estimates were imprecise, with wide confidence intervals, and should be considered exploratory. While nociception monitoring technologies such as the NOL index may help characterize intraoperative antinociception, evidence linking their use to improved postoperative outcomes remains limited [14, 15] .

The incidence of PONV was similar between groups; however, interpretation of these findings is limited by the unequal antiemetic prophylaxis between groups, which may have attenuated potential differences attributable to the anesthetic technique. Furthermore, the study was not powered to detect differences in this secondary endpoint, and the results should therefore be interpreted with caution.

Adverse events were recorded only if they occurred during routine clinical care and were not assessed through a predefined systematic protocol. The absence of systematically collected safety endpoints precludes any meaningful conclusions regarding the safety profile of OFA. In addition, outcomes potentially influenced by opioid exposure, such as respiratory events, oversedation, or length of hospital stay, were not systematically collected, further limiting comparative safety assessment.

From a broader perspective, opioid-free anesthesia may support a more individualized and multimodal approach to perioperative analgesia while reducing opioid exposure, particularly in high-risk populations such as patients with severe obesity. These strategies align with ERAS principles and current efforts to minimize opioid-related adverse effects without compromising analgesic efficacy. Although OFA has been proposed as a strategy to reduce opioid exposure, robust evidence demonstrating long-term benefits remains limited, with most available data focusing on early postoperative outcomes.

The present findings should be interpreted within the context of the exploratory pilot design. The absence of a formal sample size calculation, the single-center setting, and the unequal antiemetic prophylaxis between groups limit causal inference and may have influenced secondary outcomes. Furthermore, the limited availability of NOL monitoring and the lack of systematic safety assessment preclude definitive conclusions regarding intraoperative nociception or comparative safety profiles. Nevertheless, pilot trials are intended to generate clinically interpretable effect estimates rather than definitive evidence. In this context, the consistency of adjusted and unadjusted findings, together with the observed reduction in rescue analgesia, is consistent with a clinically relevant signal and provides estimates to inform the design and feasibility of future adequately powered confirmatory trials.

## Limitations

This study has several limitations. First, this was an exploratory pilot trial without a formal a priori sample size calculation, which may have increased the risk of type II error and limited the ability to detect differences in some secondary outcomes. The sample size was intended to provide preliminary effect size estimates to inform the design of future confirmatory studies.

Second, the single-center design and the absence of blinded outcome assessment may limit generalizability and introduce potential performance and detection bias, particularly for subjective outcomes such as postoperative pain and PONV. This lack of blinding may also have introduced measurement bias in outcome assessment.

Third, differences in antiemetic prophylaxis between groups represent an important source of bias for PONV outcomes. The more intensive regimen used in the OBA group (dexamethasone, ondansetron, and droperidol), compared with dual prophylaxis in the OFA group (dexamethasone and ondansetron), reflected routine clinical practice but may have attenuated potential differences between anesthetic strategies. Future studies should adopt standardized antiemetic protocols across groups to allow more accurate comparisons.

Fourth, NOL monitoring was available in only 41.7% of patients due to practical limitations in monitor and sensor availability, and analgesic management was not guided by NOL values. Therefore, findings related to intraoperative nociception should be interpreted as exploratory.

Fifth, adverse events were recorded only if they occurred during routine clinical care, without a predefined systematic safety assessment protocol. No structured data collection was performed for clinically relevant safety endpoints, including bradycardia, hypotension, oxygen desaturation, oversedation, respiratory events, or length of hospital stay. The absence of systematically collected safety endpoints precludes any meaningful conclusions regarding the comparative safety profile or hemodynamic effects of OFA.

Finally, the trial was retrospectively registered prior to manuscript submission, which may raise concerns regarding protocol transparency; however, all primary and secondary outcomes were predefined prior to data analysis, reducing the risk of selective reporting.

## Conclusion

In this exploratory randomized pilot trial, opioid-free anesthesia was associated with modest reductions in postoperative pain and a clinically meaningful decrease in the need for rescue analgesia compared with opioid-based anesthesia. Although absolute differences in pain intensity were small, the reduction in additional analgesic requirements suggests a potential clinical benefit. These findings should be interpreted in the context of the study’s limitations, including the small sample size, single-center design, and potential residual confounding. Overall, opioid-free anesthesia may represent a promising opioid-sparing strategy within multimodal perioperative care pathways; however, these results are hypothesis-generating and require confirmation in larger, adequately powered multicenter trials.

## Data Availability

The datasets generated and analysed during the current study are available from the corresponding author on reasonable request.
